# Rapid hearing threshold assessment with modified auditory brainstem response protocols in dogs

**DOI:** 10.3389/fvets.2024.1358410

**Published:** 2024-03-06

**Authors:** Axel Stanger, Gesine Buhmann, Stefanie Dörfelt, Yury Zablotski, Andrea Fischer

**Affiliations:** Centre for Clinical Veterinary Medicine, Ludwig-Maximilians-Universität München, Munich, Germany

**Keywords:** ABR, BAER, hearing test, chirp, ear disease, otitis, hearing loss, deafness

## Abstract

**Introduction:**

Auditory brainstem response (ABR) is the gold standard for hearing testing in dogs. ABR is commonly used in puppies to diagnose congenital sensorineural deafness. Long test times limit the use for a more comprehensive hearing screening in veterinary practice. This study aimed to establish a super-fast hearing screening protocol in dogs.

**Methods:**

Hearing thresholds were routinely measured with a mobile device designed for newborn hearing screening in 90 dogs. We introduced modifications of the ABR protocol, e. g., a binaural test mode, higher stimulus rates, a broadband chirp stimulus, and an algorithm for automatic peak V detection in a stepwise fashion. Hearing thresholds were then measured with fast protocols utilizing either 30 Hz click or 90 Hz broadband chirp stimuli with 80, 60, 40, 30, 20, 10, 0 and −10 dBnHL stimulation intensities. Interrater reliability, agreement between click and chirp hearing thresholds and correlations with clinical characteristics of the dogs were assessed.

**Results:**

Using all innovations, the test time for hearing threshold assessment in both ears was reduced to 1.11 min (mean). The chirp stimulus accentuated both, peak V and the subsequent trough, which are essential features for judgement of the hearing threshold, but preceding peaks were less conspicuous. Interrater reliability and agreement between click and chirp hearing threshold was excellent. Dogs >10 years of age and dogs with abnormal hearing score or otitis score had significantly higher hearing thresholds than younger dogs (*p* ≤ 0.001) or dogs without abnormalities (*p* < 0.001).

**Conclusion:**

The results demonstrate that modifications in ABR protocols speed-up test times significantly while the quality of the recordings for hearing threshold assessment is maintained. Modified ABR protocols enable super-fast hearing threshold assessment in veterinary practice.

## 1 Introduction

Hearing is one of the 5 basic senses in both species, humans and dogs. Hearing is essential for social interaction, orientation, and hazard prevention (1, 2). Hearing loss and deafness usually result from peripheral hearing loss, which can be divided into several categories: (A) inherited or acquired, (B) congenital or later onset, and (C) sensorineural or conductive hearing loss (3). “The most commonly observed forms in dogs are inherited congenital sensorineural, acquired later-onset sensorineural (e.g., ototoxicity, noise-induced, presbycusis) and acquired later-onset conductive (e.g., otitis externa/media)” (3).

For a long time, there were only behavioral tests to assess hearing in dogs (4, 5). The situation changed with the birth of an electrodiagnostic testing method in the late 1970s (6, 7). The auditory brainstem response (ABR), or brainstem auditory evoked response has become the most widely used hearing test in the previous decades. Among the many advantages of hearing testing with ABR are objectivity, simplicity, safety, reliability, sensitivity, and cost-effectiveness (6, 7). Furthermore, ABR can independently assess hearing for each ear. Until today, ABR is the gold standard for objective and quantitative hearing screening in newborn humans (8) and dogs (9). Examples of ABR use in veterinary practice include hearing testing in puppies for unilateral or bilateral congenital sensorineural deafness (3, 6, 10–12), in elderly dogs for age-related hearing loss (13–15), or in dogs with otitis (16–19). Furthermore, ABR has also been used in veterinary neurology to examine brainstem function (6, 20–22), as the latencies and amplitudes of the later peaks reflect conduction within the brainstem auditory pathway, and, lastly, also for the confirmation of brain death (20, 22).

In 2011, Wilson et al. (6, 7) already noted that the conventional ABR is limited in its use in veterinary medicine due to relatively long test times. The authors pointed out that that a basic hearing screening, where only a single ABR waveform might be recorded for each ear at high stimulation intensities, takes a few minutes. In contrast, a more comprehensive diagnostic and hearing threshold assessment, where dozens of ABR waveforms might be recorded for each ear, could last much longer (6, 7). Assessment of hearing threshold requires repeated testing at decreasing stimulus intensities. The hearing threshold correlates with the disappearance of peak V at low stimulus intensities.

There have been attempts to speed-up examination times for hearing screening in newborns with advanced stimulation and detection methodologies, e.g., novel binaural stimulation modes and algorithms for automated peak detection (23). Only few studies described ABR following binaural stimulation in dogs (24–26). However, these examiners recorded one ABR waveform for both ears, and thus failed to obtain independent recordings for the left and right ear. In the past and especially nowadays, more authors are using higher stimulus rates in animals (7, 9, 11, 27). This approach can also shorten test times while maintaining the test quality (7). Additionally, a relatively new type of stimulus, called “chirp,” is on the rise in human medicine (23). The chirp stimulus is known for compensating the basilar membrane dispersion and leading to a synchronous stimulation of the outer and inner hair cells in the cochlea (28–31). Thus, chirps synchronize auditory nerve fiber excitation along the length of the cochlear spiral. In contrast, the conventional click stimuli initiate synchronized responses mainly from basal auditory nerve fibers. Thus, chirp stimuli result in a higher evoked response, which is reflected in higher amplitudes of the ABR, especially in the amplitude of peak V and in improved signal-noise ratios (28–31). Up to now, there are only few reports on chirp stimuli in animals, e.g., rats (32), barn owls (33), and one study in dogs (34).

The aim of this study was to speed-up hearing threshold assessment in dogs using novel next-generation ABR methods.

## 2 Materials, equipment and methods

The animal studies were approved by the ethics committee of the veterinary faculty of LMU Munich (AZ 333-20-09-2022). The studies were conducted in accordance with the local legislation and institutional requirements. Written informed consent was obtained from the owners for the participation of their animals in this study.

### 2.1 Study design

ABR data were collected from 90 client-owned dogs from 08-2021 to 08-2023 with a next-generation ABR newborn screening module in routinely sedated or anesthetized dogs. Dogs presented either specifically for hearing testing or hearing testing was part of the diagnostic work-up. Alternatively, it was offered to the dog owner as an additional test together with other diagnostic procedures in anesthesia. All patient owners were informed about the hearing test and gave their consent for the hearing test to be carried out. During the study, consecutive modifications of the standard ABR test protocol were introduced to shorten test time (optimization phase). The impact of the modifications on waveform morphology, test time and hearing threshold was assessed. Thereafter, the relationship between the measured hearing threshold and clinical parameters, e.g., hearing score, otitis score, or age was investigated (clinical phase).

### 2.2 Sedation or anesthesia

ABR examinations were routinely performed on sedated (27 dogs) or anesthetized dogs (60 dogs). Three dogs were assessed for the confirmation of brain death. Routine protocol for sedation was butorphanol and dexmedetomidine given either IM or IV. Routine protocol for general anesthesia was propofol induction IV and maintenance with inhalation of sevoflurane or isoflurane.

### 2.3 Equipment

All measurements were performed with the mobile device Cubaudio (Article No. 100360-CUB), insert earphones and MIRA evaluation software (Supplementary File S1). The device is manufactured by Path Medical GmbH, Germering, Germany and distributed for veterinary use by Dr. Ing. Hans Oswald Ingenieurdienstleistungen, Oberpframmern, Germany, info@oexing.de in line with current EU regulations (Supplementary File S2). ABRs were recorded by subdermal stainless steel needle electrodes (12 × 0.40 mm; Natus Europe GmbH, Planegg, Germany).

### 2.4 Settings

#### 2.4.1 Electrode placements

**Figure 1:**
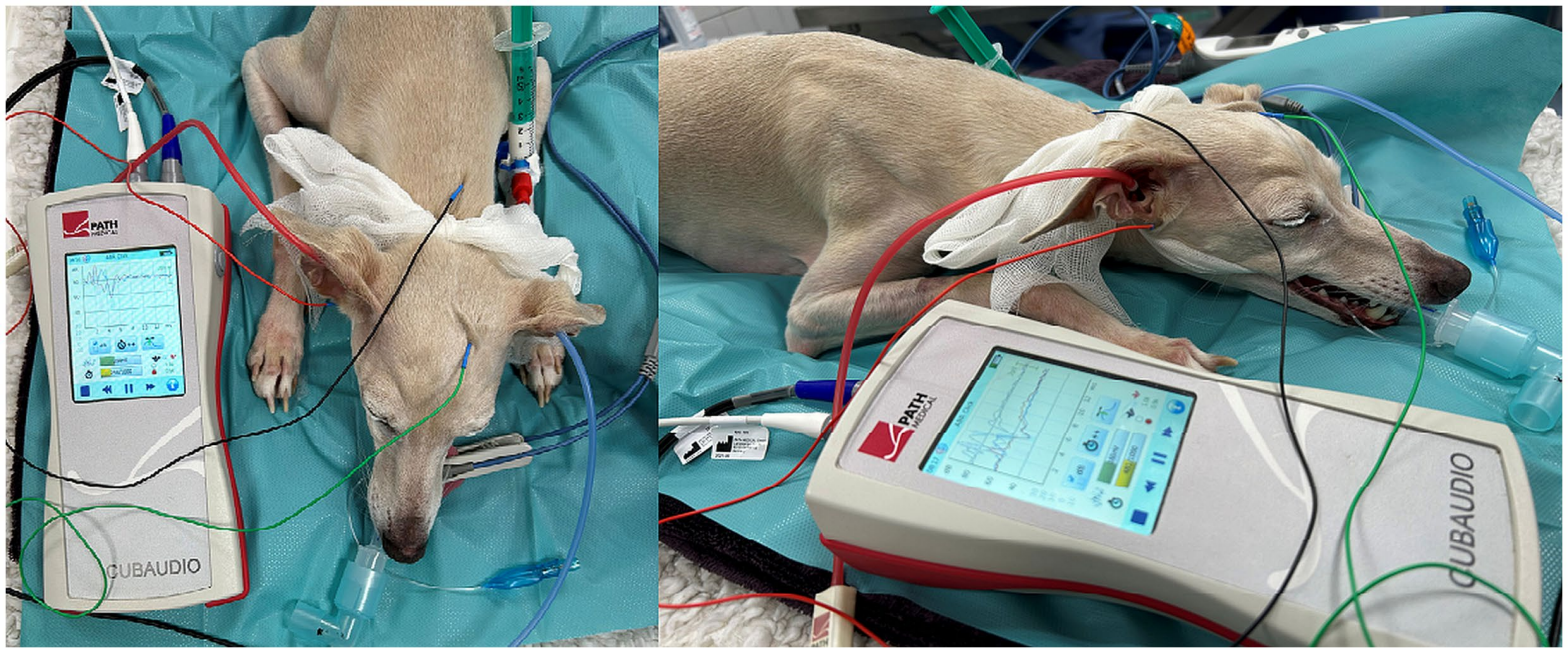
ABR hearing threshold assessment (binaural test mode). The red and blue insert headphones were placed in the right and left external ear canal, respectively. Electrode placement: inverting electrode (red) under the tragus of the right ear, non-inverting electrode (green) at the vertex and ground electrode (black) at the neck.

In the monaural test mode, the subdermal electrodes were placed as follows: inverting electrode (−) under the tragus of the measured ear (left or right ear), non-inverting electrode (+) at the vertex and ground electrode at the neck. In binaural test modes, the inverting electrode (−) was always placed under the tragus of the right ear (Figure 1). Therefore, in binaural test mode, the ABR of the right ear was recorded ipsilateral and the ABR of the left ear was recorded contralateral.

#### 2.4.2 Stimuli

Click or chirp stimuli (broadband, alternating polarity, duration 0.1 ms) were presented via insert earphones with a stepwise decrease in stimulus intensity starting at 80 dBnHL for each ABR protocol. ABR was recorded for each stimulus intensity. Averaging was performed for 1,000 stimuli per intensity (80, 60, 40, 30, 20, 10, 0 and −10 dBnHL) and correspondingly fewer stimuli when automatic peak V detection was activated. The high pass filter was preset to 80 Hz and the artifact threshold was preset to 5 μV. Masking noise at 30 dBnHL below stimulation intensity was applied to the non-tested ear in the monaural test mode. No masking noise was applied in the binaural test modes.

#### 2.4.3 Hearing threshold

The hearing threshold was defined as the lowest stimulation intensity with an identifiable peak V, as described previously in veterinary medicine (35–37). Dogs without a recordable peak V at 80 dBnHl were considered deaf and were noted with a hearing threshold of 90 dBnHL.

#### 2.4.4 ABR protocols

The ABR was recorded for 15 milliseconds after each stimulus. One thousand responses were routinely averaged for each stimulation intensity unless automated peak detection was activated. ABR hearing thresholds were assessed with three different modifications of the conventional monaural ABR protocol. The ABR protocols differed by type of stimulus (broadband click or chirp), frequency at which the stimulus was presented (11 Hz, 30 Hz, and 90 Hz) and mode of stimulation (monaural or binaural). The mode of stimulation also reflected the mode of recording. The monaural test mode only recorded an ABR for one ear and the binaural test mode recorded two individual ABRs for both ears simultaneously. Results were displayed with different colours, i.e., red, right ear; blue, left ear.

### 2.5 Optimization phase

Comparison points were the test time and the morphology of the ABR for each protocol. Modified ABR protocols were introduced during the study in a stepwise fashion: part 1: initially, we measured hearing thresholds using the click 11 Hz monaural protocol (8 dogs), followed by the click 11 Hz binaural protocol (6 dogs). Thereafter, we applied higher rates of stimulation with the click 30 Hz binaural protocol (43 dogs). Part 2: we introduced the chirp 90 Hz binaural protocol with a broadband chirp stimulus and even higher rates of stimulus presentation (90 Hz) (42 dogs). Part 3: we explored the option to carry out ABR testing with an automatic peak V detection with the click 30 Hz binaural protocol (35 dogs) and chirp 90 Hz binaural protocol (33 dogs). The algorithm was designed to detect peak V of the canine ABR. When the algorithm recognized an electrophysiologic response, e.g., peak V at the expected latency, with a sufficiently high certainty based on the signal-noise ratio, it automatically switched to the next lower stimulation intensity. Therefore, fewer repetitions were needed at higher stimulation intensities in normal hearing dogs. The algorithm for automatic peak V detection was repeatedly adjusted to the canine ABR during the study.

### 2.6 Clinical phase

Otitis externa scores (38) and hearing scores (18) were obtained for 80 dogs before ABR testing. Based on an otoscopic examination, each ear was judged for the presence of otitis externa (grade 0–12). Hearing score was obtained with a previously validated questionnaire, answered by the owner (grade 0–8). The hearing score which the dog owner provided was used for both ears. Otitis externa scores ≥4 indicated the presence of otitis externa. Hearing scores ≥2 reflected presumed hearing loss by the owner. Hearing thresholds were measured with the click 30 Hz binaural protocol. Ears without a recordable peak V at 80 dBnHL were considered deaf and were noted with a hearing threshold of 90 dBnHL.

### 2.7 Comparison of hearing thresholds and interobserver reliability

Hearing thresholds were compared between the two ABR protocols, the click 30 Hz binaural and the chirp 90 Hz binaural protocol (80 ears).

Interobserver reliability was assessed between two independent raters. Two blinded observers (AS and GB) independently assessed the ABR recordings of 22 ears and noted the respective hearing thresholds.

### 2.8 Statistical evaluation

Mean test times and confidence intervals of different ABR protocols were compared using Welch ANOVA. Games-Howell post-hoc tests compared the mean test times pairwise for the different protocols. The test times and the hearing thresholds of the click 30 Hz binaural and the chirp 90 Hz binaural protocol were compared with linear mixed effect models. In these models, estimated marginal mean values and confidence intervals were provided. Interobserver reliability between two independent raters was evaluated using Kendall’s tau. The comparison point was the noted hearing threshold. Kendall’s Tau correlation analysis indicated the degree of correlation: no correlation (*R* = 0.0 < 0.1), low correlation (*R* = 0.1 < 0.3), middle correlation (*R* = 0.3 < 0.5), high correlation (*R* = 0.5 < 0.7) and very high correlation (*R* = 0.7 < 1). The impact of an elevated hearing score, otitis score, or age on hearing thresholds were explored with a Kruskal–Wallis-test and Dunn Bonferroni post-hoc tests. *p* < 0.05 was considered significant, and the confidence level was 95% for all tests. *p*-values were adjusted for multiple comparisons by the Holm method. The raw data were descriptively analyzed in Microsoft Excel 2023 (Version 16.74, Microsoft Corporation, Redmond, WA, United States). Statistical analyses were conducted in R statistical software 2023 (Version 4.3.1, The R Foundation for Statistical Computing, Vienna, Austria) and in DATAtab Team 2023 (DATAtab: Online Statistics Calculator, DATAtab e.U. Graz, Austria).

## 3 Results

Ninety dogs (56 male, 34 female, mean age 4.3 years, range 8 weeks–15.2 years) and 175 ears participated in hearing testing. The three most common breeds were Crossbreed dogs (*n* = 16), Labrador Retrievers (*n* = 8) and Australian Cattle dogs (*n* = 8). Forty-nine dogs presented for neurologic disease, 27 dogs for hearing screening, 3 dogs for ear disease, 3 dogs for the assessment of brain death and 8 dogs for other reasons. ABRs were recorded from 153 ears and were absent in 22 ears.

### 3.1 Optimization phase part 1—introduction of binaural test mode and faster stimulus rates

#### 3.1.1 Click 11 Hz monaural protocol (reference protocol) vs. click 11 Hz binaural protocol

The first optimization point was the introduction of the binaural technique enabling simultaneous and independent recordings from both ears. The algorithm displayed ABR recordings from the right and left ear as separate waveforms distinguished by color: red (right ear), blue (left ear). The conventional click 11 Hz monaural protocol, which stimulated and recorded each ear separately, was compared to the click 11 Hz binaural protocol.

##### 3.1.1.1 ABR morphology

**Figure 2:**
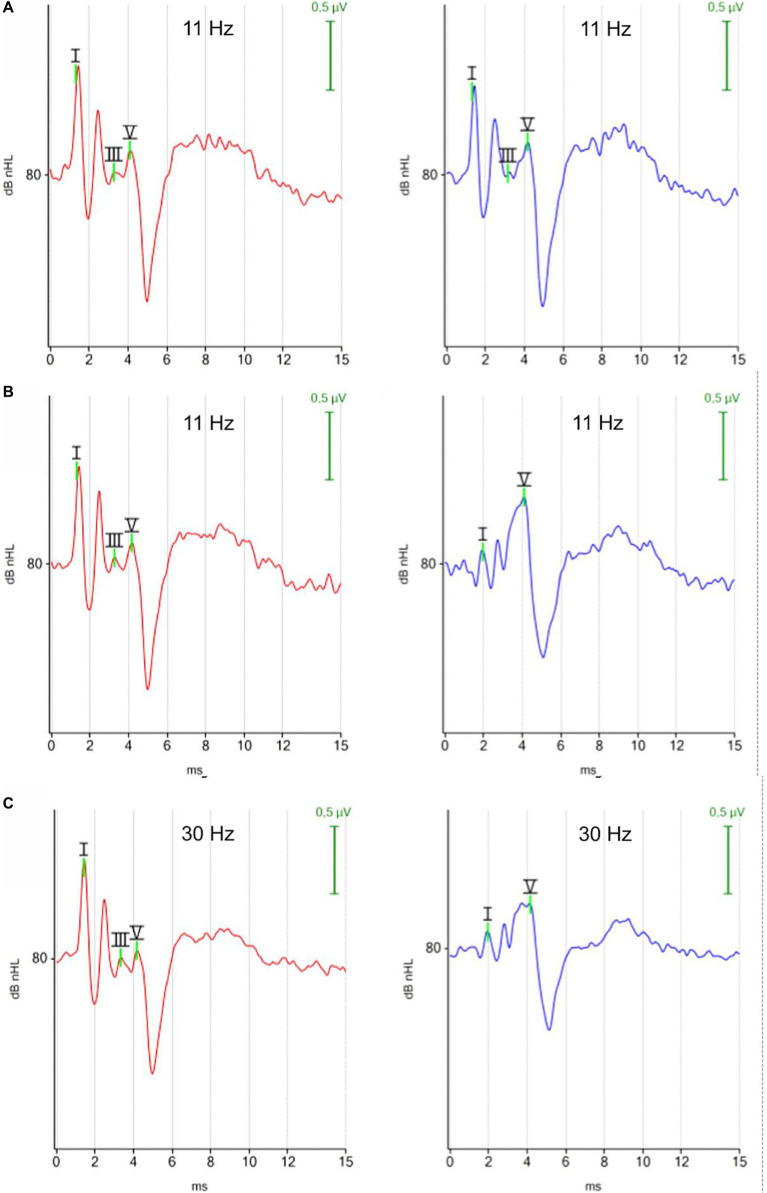
ABR waveforms with different test protocols (red, right ear; blue, left ear). **(A)** Click 11 Hz monaural. **(B)** Click 11 Hz binaural. **(C)** Click 30 Hz binaural. Each ABR waveform represents the averaged response of 1,000 repetitions at 80 dBnHL. **(A)** Monaural test mode: the position of the negative recording electrode was under the tragus of the right ear when the right ear was tested and under the tragus of the left ear when the left ear was tested. White noise was presented to the contralateral ear. The ABR waveforms exhibit minimal disparity between the right and left ear. **(B,C)** Binaural test mode: the negative recording electrode was always placed under the tragus of the right ear. The binaural test mode recorded independent ABR responses of the right and left ear simultaneously. The contralateral ABR, recorded from the left ear (blue) stands out due to its clear appearing peak V, while preceding peaks appear with reduced amplitudes.

There was no visible difference in the morphology of the ABR of the right and left ear when tested with the monaural protocol (Figure 2A). In contrast, the ABR of the left ear showed smaller amplitudes of peak I, II and III with the binaural protocol (Figure 2B). The identification of the earlier peaks of the ABR was more challenging with the binaural mode because the earlier peaks I, II, and III were viewed from a distant contralateral site, while peak V was still easily identified by its high amplitude and subsequent trough. The difference in ABR appearance between the right and left ear reflected that the ABR from the right ear was recorded with an ipsilateral montage (the stimulated ear was close to the inverting electrode), while the ABR from the left ear was recorded with a contralateral montage (the stimulated ear was far from the inverting electrode) when the binaural test mode was applied, testing both ears simultaneously.

##### 3.1.1.2 Test time

**Figure 3:**
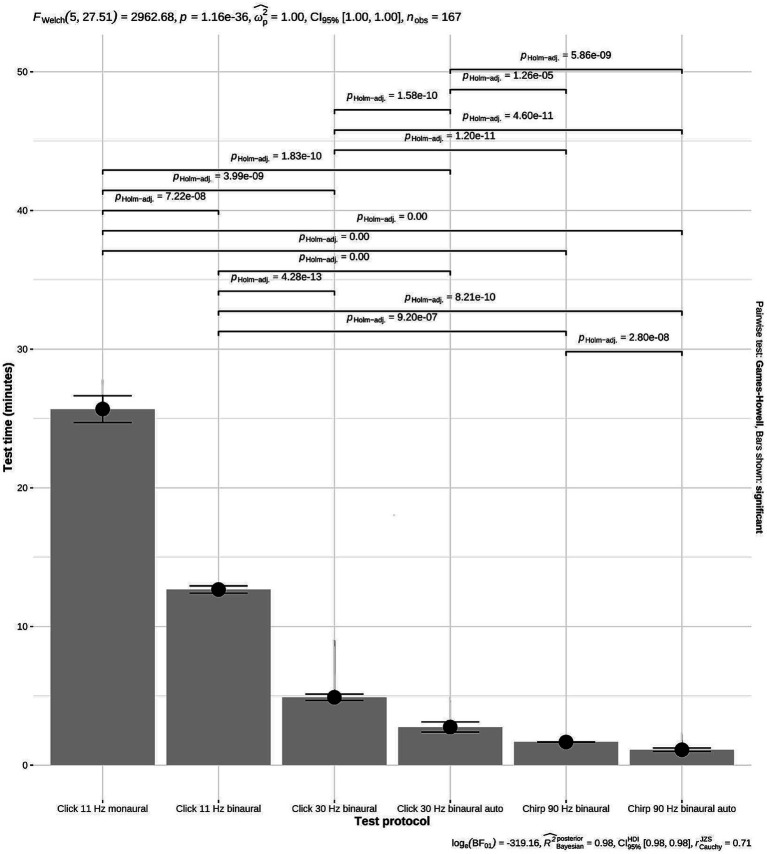
Reduction of test time with different ABR test protocols. The grey bars represent the mean test time for measuring hearing thresholds in both ears. The test time decreased significantly with the introduction of new protocols from 25.7 min to 1.11 min (mean, *p* = 1.16 × 10^−36^; Welch ANOVA). Black dot: mean values; horizontal lines: 95% confidence intervals (CI). Click 11 Hz monaural (*n* = 8): 25.7 min, 95% CI 24.7–26.6; click 11 Hz binaural (*n* = 6): 12.7 min, 95% CI 12.4–12.9; click 30 Hz binaural (*n* = 43): 4.89 min, 95% CI 4.66–5.12; click 30 Hz binaural incl. automatic peak V detection (*n* = 35): 2.75 min, 95% CI 2.38–3.11; chirp 90 Hz binaural (*n* = 42): 1.67 min, 95% CI 1.66–1.68; chirp 90 Hz binaural incl. automatic peak V detection (*n* = 33): 1.11 min, 95% CI 0.98–1.24.

The option to test binaurally resulted in significantly reduced test times (*p* = 7.22 × 10^−8^) (Figure 3). Measurement of the hearing thresholds with a click 11 Hz monaural protocol (80, 60, 40, 30, 20, 10, 0, −10 dBnHL) took 12.85 min (mean) for each ear and 25.7 min for both ears (mean, 95% CI 24.7–26.6 min) when standardized averaging (1,000×) was used for each tested stimulus intensity. The equivalent binaural protocol (click 11 Hz binaural) saved about half of the test time, with a mean test time of 12.7 min for both ears (95% CI 12.4–12.9 min). There was also no need to reposition electrodes with the binaural test mode which saved additional manipulations and test time.

#### 3.1.2 Click 11 Hz binaural protocol vs. click 30 Hz binaural protocol

The next optimization point was the introduction of increased stimulus rates.

##### 3.1.2.1 ABR morphology

There was no visible difference in the morphology of the ABR waveforms when the stimulus rate was increased from 11 Hz to 30 Hz (Figures 2B,C).

##### 3.1.2.2 Test time

The time savings of the click 30 Hz binaural protocol were immense. The click 30 Hz binaural protocol required 4.89 min (mean, 95% CI 4.66–5.12 min) for hearing threshold assessment in both ears and saved more than half of the test time (*p* = 4.28 × 10^−13^; Figure 3).

### 3.2 Optimization phase part 2—introduction of a new chirp stimulus with 90 Hz stimulus rate

#### 3.2.1 Click 30 Hz binaural protocol vs. chirp 90 Hz binaural protocol

This part introduced the new chirp stimulus with an even higher stimulus rate of 90 Hz. The points of comparison included morphology, test time and hearing threshold.

##### 3.2.1.1 ABR morphology

**Figure 4:**
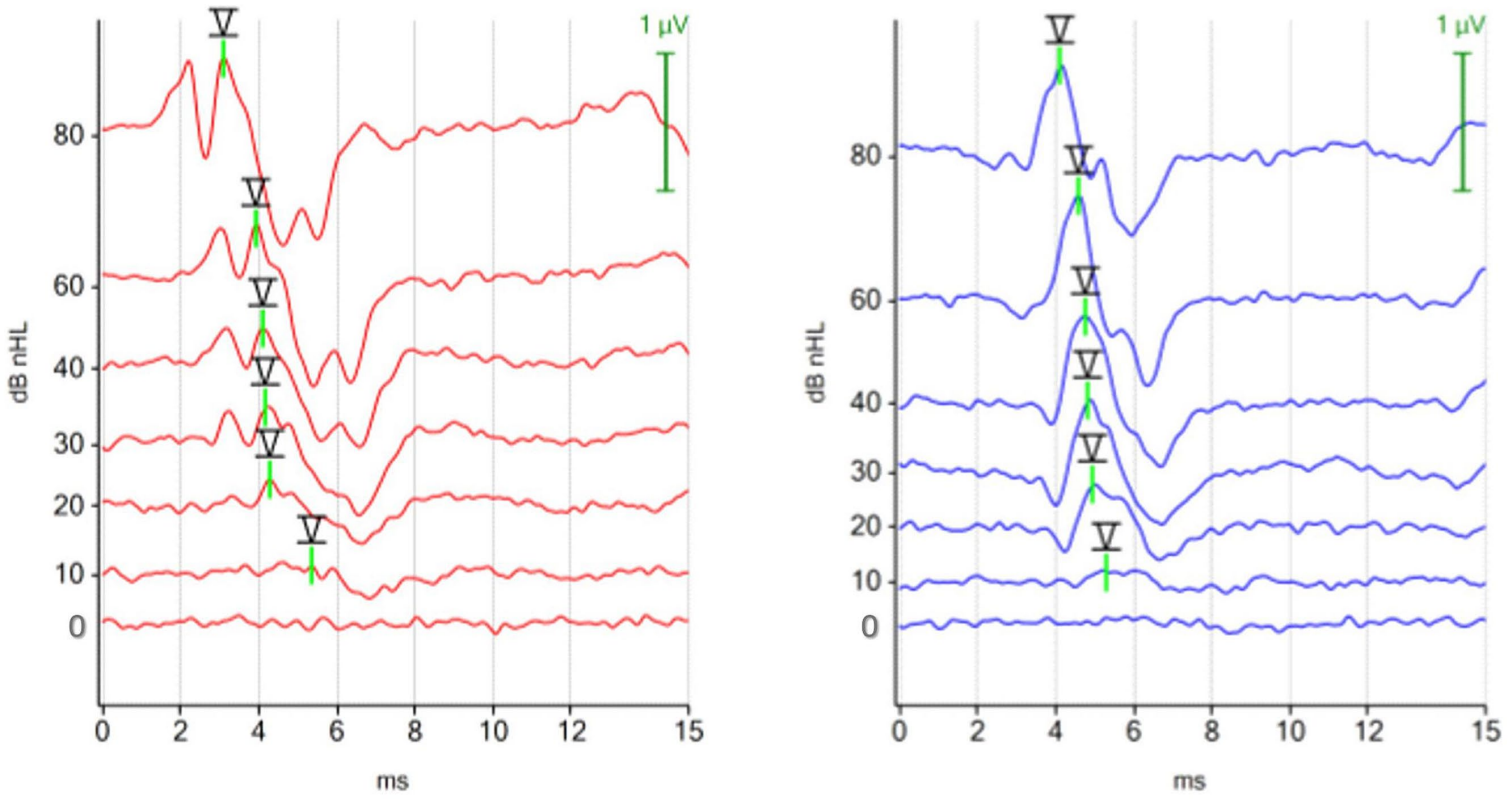
ABR hearing threshold assessment with the 90 Hz chirp binaural protocol. The figure shows multiple ABR waveforms generated at different stimulation intensities (mean test time 100 s; hearing threshold in both ears 10 dBnHL). The ipsilateral (red, right ear) and the contralateral (blue, left ear) ABR waveforms were focused on peak V and the subsequent trough. Preceding peaks are less visible in the contralateral recorded ABR (blue, left ear).

The waveforms of the ABR recorded with a chirp 90 Hz differed notably from the waveforms of the ABR recorded with a click 30 Hz stimulus. Using the 90 Hz chirp, the ABR from the right ear (red) displayed two identifiable peaks before the characteristic trough after peak V, while the ABR from the left ear (blue) showed only one impressive peak before the trough. In contrast, previous peaks were easier to identify with the 30 Hz click binaural protocol. The ABR waveform of the chirp suggested optimization primarily for peak V with its following trough, while previous peaks were visualized to a limited extent (Figure 4).

##### 3.2.1.2 Test time

The test time for measuring hearing thresholds in both ears decreased from 4.89 min (mean, 95% CI 4.66–5.12) with the click 30 Hz protocol to 1.67 min (mean, 95% CI 1.66–1.68) with the chirp 90 Hz protocol (*p* = 1.20 × 10^−11^) (Figure 3).

##### 3.2.1.3 Hearing thresholds

The hearing thresholds were compared in 80 ears. 72.5% (58/80) did not differ, 22.5% (18/80) differed by 10 dBnHL and 5% (4/80) differed by 20 dBnHL. The estimated marginal mean hearing threshold calculated using linear mixed effect models was 19 dBnHL (95% CI 13–24 dBnHL) for the click 30 Hz binaural protocol, and 20 dBnHL (95% CI 14–25 dBnHL) for the chirp 90 Hz binaural protocol.

### 3.3 Optimization phase part 3—exploration of automatic peak V detection

#### 3.3.1 Click 30 Hz binaural protocol and chirp 90 Hz binaural protocol with automatic peak V detection

This test phase explored the additional functionality of a newly developed algorithm for automatic peak V detection in the canine ABR.

##### 3.3.1.1 Test time

The algorithm for automatic peak V detection achieved additional time savings. The test time for assessment of hearing thresholds in both ears decreased to 2.75 min (mean, 95% CI 2.38–3.11 min) with the click 30 Hz binaural protocol and to 1.11 min (mean, 95% CI 0.98–1.24 min) with the chirp 90 Hz binaural protocol (Figure 3).

### 3.4 Interobserver reliability

Interobserver reliability was excellent. Two independent raters determined 22 hearing thresholds for 11 dogs. The observers agreed in 21 of the 22 hearing thresholds (95.5%; *R* = 1; *p* = 0.018).

### 3.5 Clinical phase—relationship between ABR hearing thresholds and hearing score, otitis score or age (80 dogs, 160 ears)

**Figure 5:**
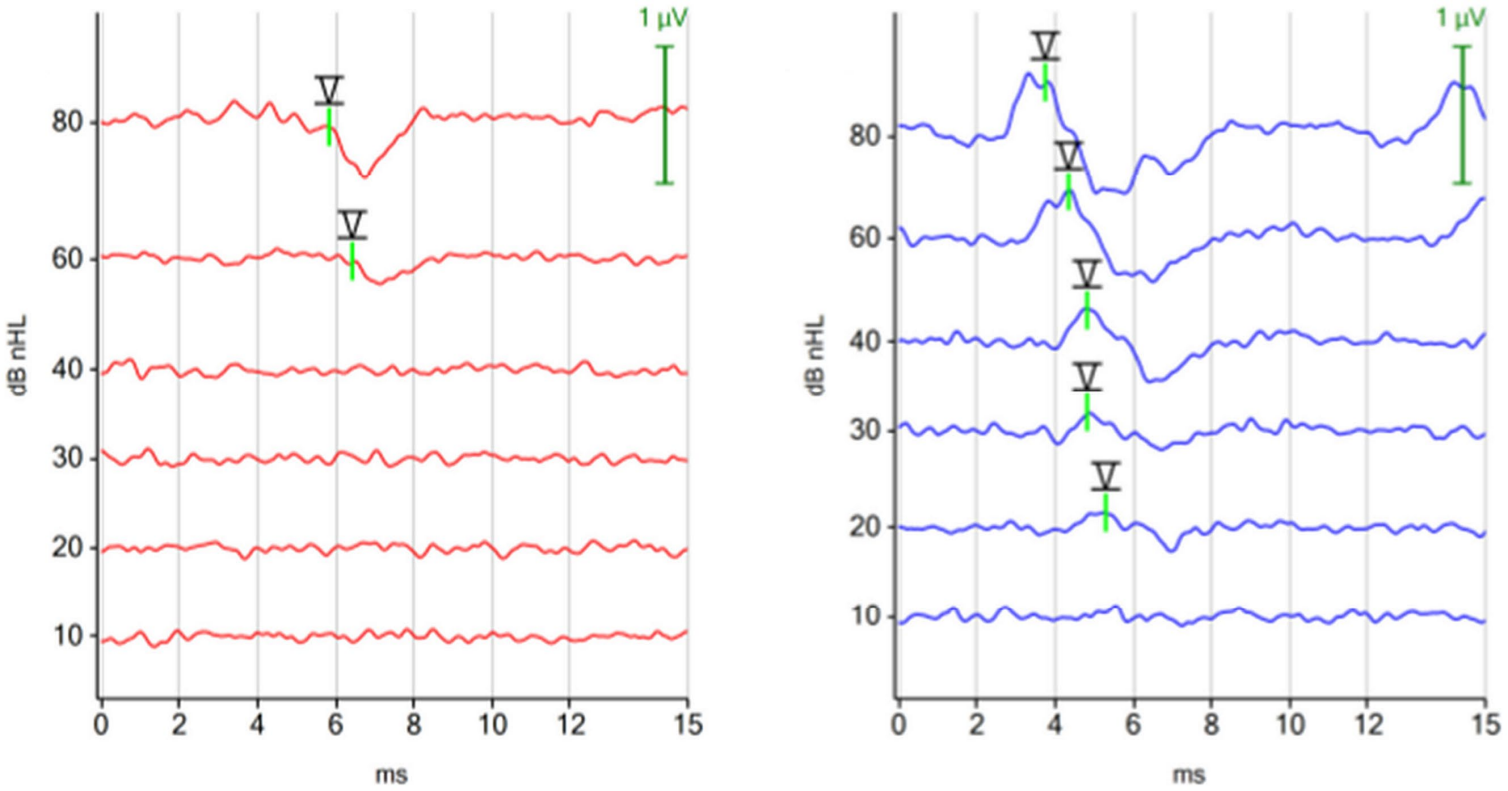
ABR hearing threshold assessment (chirp 90 Hz binaural) in a dog with otitis externa et media in the right ear due to a cholesteatoma. The left ear was not affected. Right ear (red): hearing threshold 60 dBnHL. Left ear (blue): hearing threshold 20 dBnHL.

Table 1 shows the relationship between hearing score, otitis score or age and ABR hearing thresholds. The hearing score was elevated in 40 ears (20 dogs, ≥2) and the otitis score was elevated in 25 ears (≥4). Ears with hearing loss reported by the dog owner (mean 68 dBnHL), or ears with otitis externa (mean 65 dBnHL) showed higher hearing thresholds than normal ears (mean 15 dBnHL, *p* < 0.001). Figure 5 displays the ABR of a dog with otitis externa et media in the right ear due to a cholesteatoma. The ABR shows an increase in hearing threshold in the right ear (60 dBnHL). Elderly dogs (>10 years: mean 58 dBnHL) showed higher hearing thresholds than younger dogs (<5 years: mean 26 dBnHL, *p* < 0.001; 5–10 years: mean 26 dBnHL, *p* = 0.001). The results show that clinical impairments and high age contribute to an elevated hearing threshold.

**Table 1:** Relationship between ABR hearing thresholds and hearing score, otitis score or age (80 dogs, 160 ears).

	Ears (*n*)	Hearing status		Mean hearing threshold (dBnHL)	*p*-value (compared to normal ears)	*p*-value (compared to age >10 years)
		Deaf (ht. >80 dBnHL)	Hearing (ht. ≤80 dBnHL)			
All ears	160	9.4% (15/160)	90.6% (145/160)	30		
Normal ears	111	—	100.0% (111/111)	15		
Abnormal ears	49	30.6% (15/49)	69.4% (34/49)	67	<0.001	
Hearing score <2	120	—	100.0% (120/120)	18		
Hearing score ≥2	40	37.5% (15/40)	62.5% (25/40)	68	<0.001	
Otitis score <4	135	6.7% (9/135)	93.3% (126/135)	24		
Otitis score ≥4	25	24% (6/25)	76% (19/25)	65	<0.001	
Hearing score ≥2 and otitis score ≥4	16	37.5% (6/16)	62.5% (10/16)	74	<0.001	
Age <5 years	114	7.9% (9/114)	92.1% (105/114)	26		<0.001
Age 5–10 years	22	9.1% (2/22)	90.9% (20/22)	26		0.001
Age >10 years	24	16.7% (4/24)	83.3% (20/24)	58		

Dogs with elevated hearing or otitis score and dogs >10 years had significantly higher ABR hearing thresholds than dogs with normal ears and younger dogs. ht., hearing threshold; dBnHL, decibel normal hearing level. Calculated with a Kruskal–Wallis-test and Dunn Bonferroni post-hoc tests.

### 3.6 Brain death

**Figure 6:**
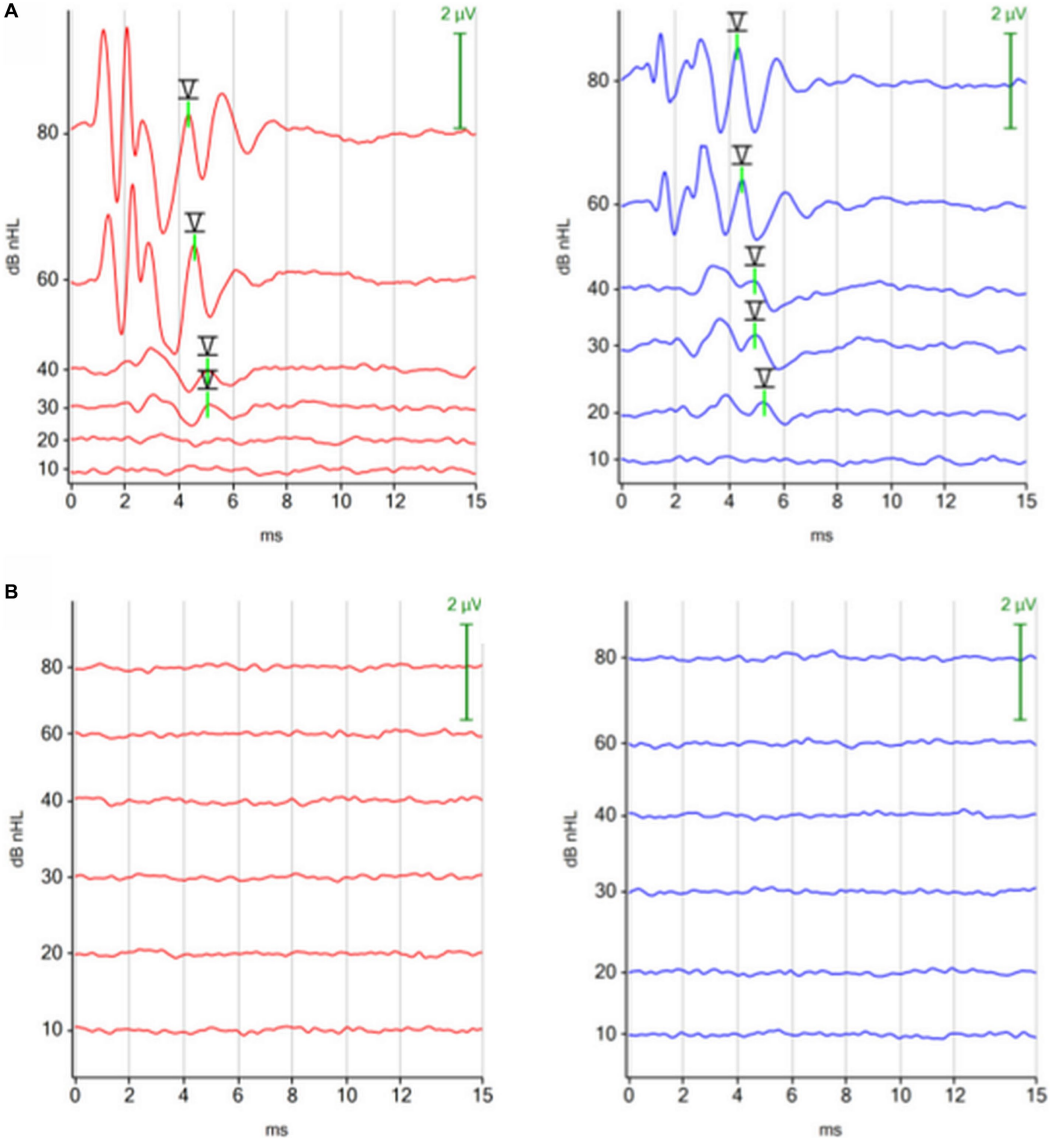
ABRs (click 11 Hz binaural) before and after respiratory arrest in a dog, which died in the postoperative phase after surgery for a retrobulbar abscess. **(A)** ABR before surgery: normal. **(B)** ABR after respiratory arrest (dog was ventilated): absent, consistent with brain death.

Using the ABR as a diagnostic tool, 3 comatose dogs were diagnosed with brain death. The reasons for brain death were: intracranial mass with acute intracranial hemorrhage, anesthesia incident, and postoperative complication after surgery. Figure 6 shows the ABRs of a dog, which died in the postoperative phase after surgery for a retrobulbar abscess.

## 4 Discussion

This study established a super-fast hearing screening protocol with a 90 Hz chirp stimulus and a binaural test mode. Hearing thresholds of both ears could be obtained within 1–2 min without the need for electrode repositioning. Altogether, this new test protocol paved the road for the routine application of hearing threshold measurements in veterinary practice, particularly for dogs with suspected ear diseases.

The study explored modifications of different ABR protocols for hearing threshold assessment in dogs. The investigation points were test time, test quality and clinical correlation. The results demonstrated that modified ABR protocols are both fast and reliable in assessing hearing thresholds.

### 4.1 Binaural stimulation/recording (binaural test mode)

The binaural test mode saved about half the test time for hearing threshold assessment for both ears. Using this mode, hearing thresholds of both ears could be measured in one measuring cycle. Monaural test modes require two measurement cycles, because each ear is tested separately. Furthermore, the conventional monaural test mode loses additional time for repositioning of the recording electrode and starting the second measurement. The ABR of the contralateral “electrode far ear” appeared slightly different than the ABR of the ipsilateral “electrode near ear” in the binaural test mode. The amplitudes of the peaks I–III were notably smaller and the identification of these peaks was more challenging in the contralateral recordings. In contrast, peak V was always easy to identify due to its more central origin, its large amplitude and subsequent trough. The binaural test mode proves sufficient for hearing threshold assessment due to the good visualization of peak V. In contrast, the monaural test mode should be preferred when the ABR is used as a diagnostic tool for assessment of neurologic functions, i.e., the assessment of brainstem conduction within the central auditory pathway. In such instances, the analysis requires precise measurements of the peak and interpeak latencies for each ear (20, 21).

Previously, other authors already reported on binaural stimulation in dogs (24–26). However, these studies showed the ABR of both ears as a single waveform after binaural stimulation and did not provide single traces for each ear. In contrast, the Cubaudio uses advanced test protocols and algorithms which enable simultaneous recording of independent traces for each ear. In 2018, Andre Lodwig (Path Medical GmbH, Germering, Germany), explained the functionality of the binaural test mode on the company’s website (39). Lodwig emphasized that stimulus rates, presented to both ears, were not allowed to be correlated in the binaural test mode. He provided the following example: “A traditional recording scheme is to just apply different but constant stimulus rates to both ears, such as 37 Hz and 41 Hz. Even preferable choices would be stimulus rates that do not have a common period (1 Hz in the example above), such as 37.3816394 Hz and 41.136818273 Hz etc. Spectrally, any neural response that is evoked by either of the stimuli contains just the stimulus rate and multiples. This means, that if averaging is done in synch to each ear’s stimulus rate, responses can be recorded independently. The auditory evoked potential signal that is evoked from each other ear just appears as a very small added EEG noise, since it is not correlated to the averaging” (39). It is even possible to modify the stimulus rate during testing to achieve nearly equal average stimulus rates for both ears. This mode is expected to enhance robustness against artifacts (39). Binaural stimulation and recording is used in human audiology with next generation ASSR testing which evaluates several hearing thresholds for several test frequencies simultaneously for both ears (23). There are other studies in human medicine which report about binaural stimulation and binaural recording (40–42). However, the aim of these studies was not to assess individual ABR results for the right and left ear simultaneously. Instead, the authors investigated the influence of binaural stimulation, which is the binaural interaction component, on the overall measurement (40–42).

### 4.2 Higher stimulation rates

Higher stimulation rates could immensely reduce the required test time for ABR hearing threshold assessment. In the past, some authors already applied higher stimulation rates in dogs (7, 9, 11). In 2011, Wilson et al. (7) reported that the quality of click ABR waveforms could be maintained in sedated dogs when the stimulus rate was increased from 11 Hz to 33 Hz or even to 91 Hz. Our results align with Wilsons’s observations. Even, for binaural recordings, we did not appreciate any discernible difference in the morphology of the ABR waveforms by increasing the stimulus rate from 11 Hz to 30 Hz. A draw-back of the present study is that we did not investigate clicks presented with stimulation rates as high as 90 Hz.

### 4.3 New stimulus “chirp”

The chirp stimulus can compensate the basilar membrane dispersion, leading to synchronous stimulation of the hair cells in the cochlea. This synchronization results in higher compound action potentials and higher amplitudes of the evoked response, especially in the amplitude of peak V (28–31). Consequently, waveforms are easier to detect and test time can be reduced (30). In human medicine, the chirp stimulus is currently mostly utilized in frequency-specific measurements, such as the auditory steady-state responses (ASSR) or frequency-specific ABRs, replacing traditional frequency-specific tone pips or tone bursts (43–47). In 2020, Eder et al. (46) summarized various studies as follows: “A chirp is more efficient than a corresponding click in the recording of the ABR and of auditory steady-state responses (ASSR).” In our recordings in dogs, it became evident that the chirp stimulus was optimized for hearing threshold assessment. Peak V always had a high amplitude, whereas previous peaks were not reliably identified. Therefore, the chirp stimulus is not recommended for the investigation of peak and interpeak latencies. In 80 ears, in which the hearing thresholds were measured using a click and a chirp stimulus, the estimated marginal mean hearing threshold of the chirp 90 Hz binaural protocol differed only by 1 dB from the click 30 Hz binaural protocol. Given that hearing threshold assessments are usually performed in 10 dBnHL steps, this marginal deviation can be considered negligible. Furthermore, hearing thresholds were identical in 72.5% and differed by only 10 dBnHL in 22.5%. Hearing thresholds obtained with traditional stimuli and chirp stimuli appear also highly correlated in humans (23). The additional time savings and the fact, that hearing threshold assessment was possible within 1–2 min for both ears simultaneously, strongly supports the use of the superfast chirp 90 Hz binaural hearing screening protocol. Hearing threshold measurements with the chirp 90 Hz binaural protocol are sufficient for most clinical questions, in particular hearing screenings in veterinary practice. In this study, we did not compare the click 90 Hz binaural and chirp 90 Hz binaural protocol, because we just aimed to prove that the chirp 90 Hz binaural protocol is as good as the click 30 Hz protocol, but much faster for hearing threshold measurements. It should be noted that the used chirp stimulus was originally developed with a cochlea model designed for humans. Future studies using a canine-specific and dog breed-specific cochlear model may lead to even lower hearing thresholds and better automatic peak V detection, unlocking the full potential of the chirp stimulus. Then, a comparison of the standard click and multiple chirps with a stimulus rate of 90 Hz would be of interest.

### 4.4 Automatic peak V detection

The introduction of an algorithm for automatic peak V detection in the canine ABR further reduced test time. Fewer stimuli were needed to record a response at high stimulation intensities in normal hearing dogs. The test time for both ears approached 1 min when this modality was additionally used (Figure 3). The review of the ABR waveforms confirmed that the algorithm correctly detected peak V. The automatic mode averaged progressively more stimuli for each intensity, reaching up to 1,000 stimuli, when the amplitude of peak V was lower and closer to the hearing threshold. Then the examiner judged whether there was still a peak V or not. While the algorithm for automatic peak V detection was adjusted throughout the study, a limitation is that the automatic setting of the marker for peak V is not consistently precise at the highest point of the peak and some fine-tuning may still be needed for accurate placement. In human medicine, many authors emphasize the importance of automatic methods for quality assessment or automatic peak detection to reduce test time, obtain objective evaluations of results, reduce human mistakes or bias and improve test uniformity (23, 48). The need for an objective and fast hearing screening in newborns has been present for a considerable period (48). Presently, there are numerous studies in human medicine which describe ABR threshold measurements with an automatic peak or wave detection (48–51). To the best of the authors’ knowledge, there was no data about automatic peak detection in companion animals. However, in veterinary medicine, optimizing automatic wave and peak detection could introduce even more objectivity in testing.

### 4.5 Test time

In 2011, Wilson et al. (7) already reported about time savings in comprehensive diagnostic and hearing threshold assessment by increasing stimulus rates up to 91 Hz. In summary, the use of all modifications—binaural test mode, increased stimulus rate, chirp stimulus and automatic peak V detection—shortened the test time for hearing threshold measurement significantly. Each additional modification contributed to a further significant reduction of test time. Overall, the test time for hearing threshold assessment in both ears could be reduced from 25.7 min with the click 11 Hz monaural protocol to 1.11 min with the chirp 90 Hz binaural incl. automatic peak V detection protocol. This fact suggests that super-fast ABR hearing testing could be an attractive option for veterinary practitioners to offer to their clients.

### 4.6 Clinical data

The results showed that clinical impairments, such as otitis externa, or a higher age were associated with a decreased hearing ability of the dogs. Ears affected by otitis externa and dogs >10 years of age had mean hearing thresholds of 65 dBnHL and 58 dBnHL, respectively. In contrast, dogs <10 years of age and ears without abnormalities had mean hearing thresholds of 26 dBnHL and 15 dBnHL, respectively. The measured hearing thresholds correlated also with the results of the hearing loss questionnaire (18). The mean hearing threshold of dogs with an elevated hearing score (68 dBnHL) was significantly higher than in dogs with unremarkable hearing and otitis scores (15 dBnHL). All 15 deaf ears were identified by the questionnaire. There are a few other studies that investigated hearing loss in dog populations and demonstrated similar associations. In 2013, Mason et al. (18) already noted that the hearing score, provided by the dog owner, was useful in detecting grade 2 hearing loss or higher in dogs. In their study, a grade 2 hearing loss indicated bilateral hearing loss with a threshold ≥41 dBnHL. Among dogs with otitis externa or media, only 22% had normal hearing (≤25 dBnHL), 25% had unilateral hearing loss and 53% had bilateral hearing loss (18). In 2010, Harcourt-Brown et al. (17) compared the hearing threshold of Cavalier King Charles Spaniels with and without middle ear effusion. ABR testing revealed conductive hearing loss in dogs with middle ear effusion. The median hearing threshold for dogs with middle ear effusion was 60 dBnHL, compared to 30 dBnHL for dogs without middle ear effusion. Overall, these studies showed a significant relationship between the presence of otitis and a higher hearing threshold (17, 18).

In 2008, Ter Haar et al. (15) investigated the effects of aging on frequency-specific brainstem auditory-evoked responses. The thresholds of older dogs (age 11–14 years) were significantly higher at all tested frequencies than the thresholds of the two younger dog groups. Their results indicated that age-related hearing loss begins at an age of 8–10 years in dogs. More recently, in 2022, Fefer et al. (14) investigated the “Relationship between hearing, cognitive function, and quality of life in aging companion dogs” and observed that dogs with a hearing threshold of 70 or 90 dB were significantly older than those with a hearing threshold of 50 dB. These studies are also consistent with our findings and demonstrate the potential of hearing screening for the routine diagnosis of hearing disorders in dogs.

The 3 comatose dogs, which were diagnosed to be brain death, did not show any peaks in their ABRs. Previously, none of these dogs were considered to be deaf by the owner. In 1994, Steiss et al. (20) reported on 4 dogs showing signs of brain death. Two dogs did not show any peaks in their ABR. They did not report any details about the ABRs of the other 2 dogs. In humans a flat ABR is the most common pattern in brain death. Nevertheless, peak I could still be present in some patients immediately after brain death. Peak I, which arises from the auditory nerve near the spiral ganglion, disappears gradually following brain death in line with increasing hypoxia and hypothermia of the cochlea (52).

In summary, the integration of ABR modifications derived from next generation newborn hearing screening achieved a significant reduction in test time. Thus, hearing threshold measurements can be part of the routine diagnostic work-up in veterinary practice.

## Data availability statement

The raw data supporting the conclusions of this article will be made available by the authors, without undue reservation.

## Ethics statement

The animal studies were approved by Ethics Committee of the Veterinary Faculty of LMU Munich (AZ 333-20-09-2022). The studies were conducted in accordance with the local legislation and institutional requirements. Written informed consent was obtained from the owners for the participation of their animals in this study.

## Author contributions

AS: Conceptualization, Data curation, Formal analysis, Investigation, Methodology, Validation, Visualization, Writing – original draft. GB: Data curation, Investigation, Validation, Writing – review & editing, Formal analysis. SD: Investigation, Methodology, Supervision, Writing – review & editing, Conceptualization. YZ: Formal analysis, Validation, Visualization, Writing – review & editing. AF: Conceptualization, Investigation, Methodology, Project administration, Supervision, Validation, Visualization, Writing – review & editing.
