# Supplementary material for: Rapid hearing threshold assessment with modified auditory brainstem response protocols in dogs
Source: Front Vet Sci. 2024 Mar 6;11:1358410. doi: 10.3389/fvets.2024.1358410 (PMC10951061; doi:10.3389/fvets.2024.1358410)
Supplement: Supplementary file 1 [file Data_Sheet_1.pdf]

# Simple and quick as 1, 2, 3!

**The reliable but portable pet hearing screening system.**

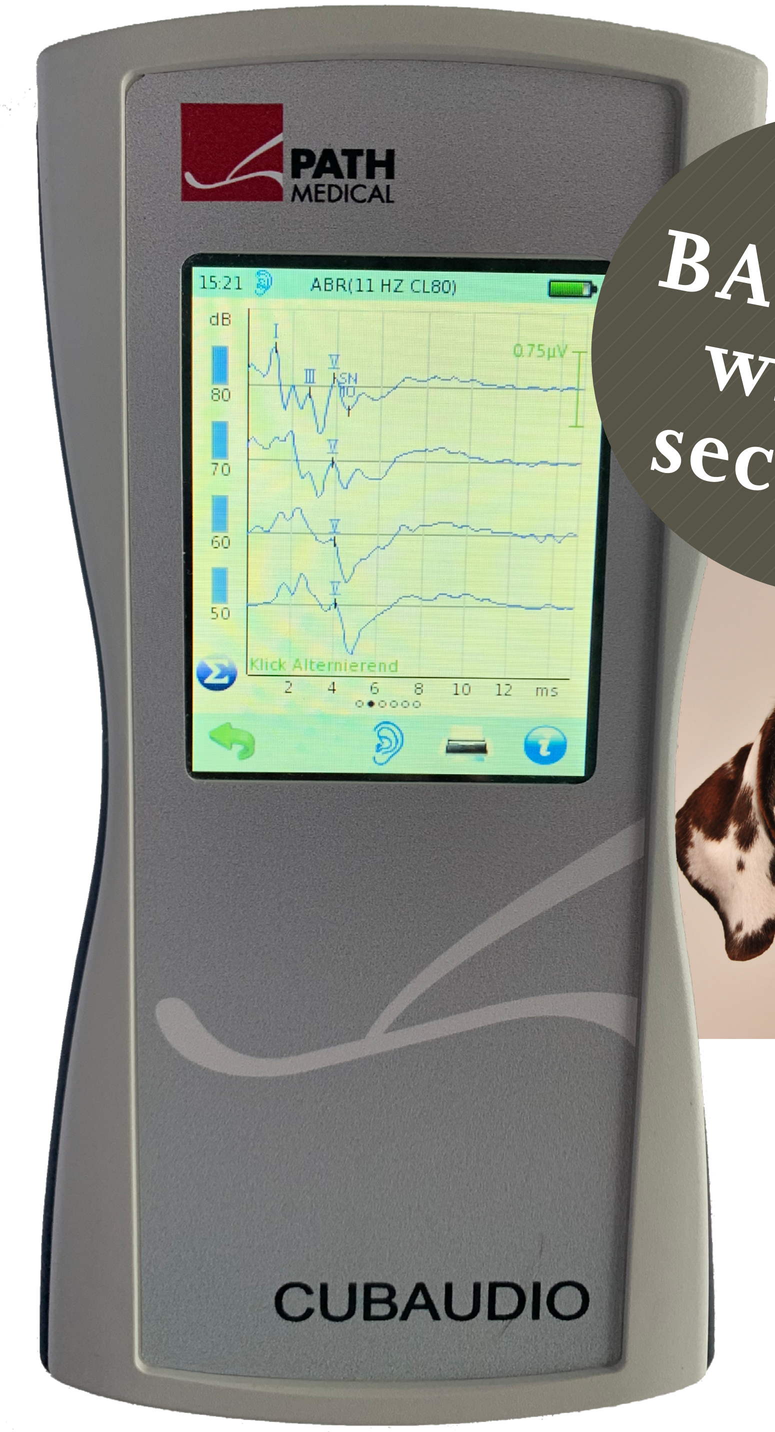


**Your BAER test within seconds.**

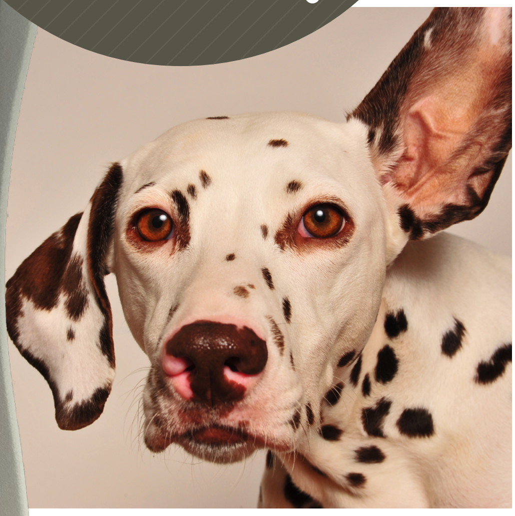

**Made in Germany**

PATH MEDICAL

**Advanced Options:**

**Automatic Stopping:** Have the test automatically stop once a satisfactory response is acquired.

**Multiple Stimulus Types**: Select from: click, chirp or tone bursts at 500, 1000, 2000 or 4000 Hz stimulus signals.

**Robust in every electric environment:** The portable, battery driven instrument is floating to other instruments and automatically detecting external sources of distortion. The impedance checker is automatically surveying the measurement process at all times.

**Amplifier Options**: Manually set the artifact rejection level or choose automated routines. Choose optimized protocols for sedated pets and try even non-sedated options if applicable.

**Customizable Display:**

Increase the variability by connecting the portable instrument via an optically insulated line via USB to your PC. Use your PC screen to display the results online and interact with the device via your mouse/touchpad as you would touch the device itself.t.

**Recording Information:** View all the acquisition information for each wave; including stimulus, rate, peak values and inter-peak latencies. Instant Signal to Noise Ratio estimation and Residual Noise calculation for all acquired recordings.

**Multi-EP Display** option: Show your recordings in choronological order, overlaid, summed up … find the hearing threshold easily without the need of time consuming postprocessing.

**ASSR and OAE Screening System:**

Upgrade your system to include FMDPOAE or TEOAE or even ASSR modules which enhance your screening capabilities to professional diagnostic capabilities. Did you know how easy it is to get an objective estimate of the hearing threshold of any pet?

**Integrated Database:**

Transfer all data to your PC and use multiple reporting options. Manage your patients easily and keep organized records of previous tests. Export your subject's information via xls or xml export for further data analysis or research activities.

**The standard BAER set includes:**

· Instrument with carrying bag
· Charger
- electrode cable
- set of needle electrodes
- insert earphones
- set of insert earphone tips
- direct print feature to print directly to pdf
· Self-Check Module

**Optionals features include:**

- OAE probe and accessories
- headphones, bone conductor
- … much much more features to use for investigational purposes

**PATH MEDICAL develops and manufactures in Germany, giving us the confidence to extend our standard warranty on manufacturing defects up to even 5 years!**

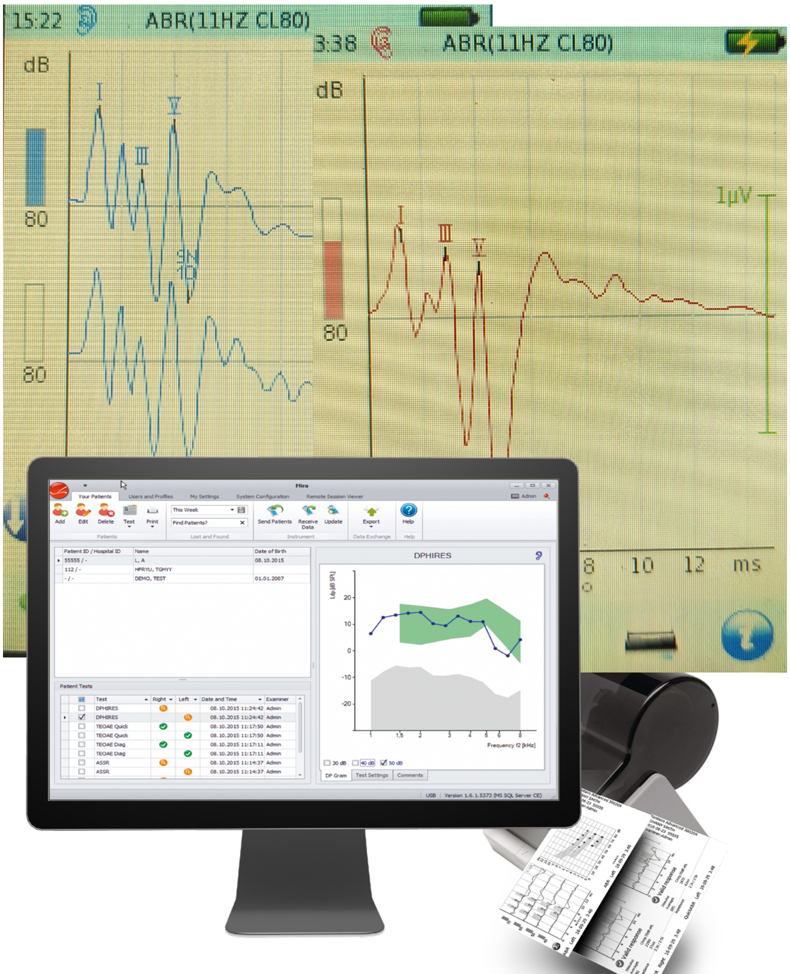


**Automatic Response Assessment:**

The software labels recordings automatically as Probable or no-response as they are acquired, based on advanced detection algorithms..

**Varied Stimulation Options:**

Select from Clicks or a variety of tones to present stimulation. Output in dB SPL.

**Accessible Online Help:**

Context sensitive help available on the device.

**Easy Data Acquisition:**

Save time and acquire data quickly, right out of the box. All necessary parameters are pre-programed in to the system, just enter the pet's information and Start.

**Customizable Protocols:**

Fast setup of screening protocols for different frequencies and intensities. Save protocol files for future use, and reuse them whenever necessary. Choose intensities, number of sweeps, stimulus type, stimulators and rate.

**Technical Specifications:**

Device dimensions: 209 x 98 x 52 mm, ca. 500 g, ca. 475 Display: 240 x 320 pixel; graphic LCD 3.5", resistive touch screen, real time-clock, piezo-electric sound generator, USB, Output voltage and nominal impedance (headphone socket): 5 Vpp, 32 Ω Power consumption: max. 2 W. Memory capacity: up to 1000 patients, ca. 1000 tests (dependent on test type).

**https://www.oexing.de/cubaudio/**

PATH MEDICAL


Manufactured by:

PATH MEDICAL GmbH

Landsberger Straße 65
82110 Germering
Germany

Exclusive distribution by:
Dr.-Ing. Hans Oswald Ingenieurdienstleistungen
Esterndorf 9
85667 Oberpframmern
Germany

Tel +49 89 38468115 info@oexing.de / www.oexing.de


100837-99_MA_CUBAUDIO_EN_01, September 2021
