# Supplementary material for: Rapid hearing threshold assessment with modified auditory brainstem response protocols in dogs
Source: Front Vet Sci. 2024 Mar 6;11:1358410. doi: 10.3389/fvets.2024.1358410 (PMC10951061; doi:10.3389/fvets.2024.1358410)
Supplement: Supplementary file 2 [file Data_Sheet_2.pdf]

PATH medical GmbH
Landsberger Str. 65
D-82110 Germering
Germany
Tel. +49 / 89 / 80076502
Email: info@pathme.de
Internet: www.pathme.de

EG-Konformitätserklärung
Declaration of Conformity

DEUTSCH

Wir

PATH medical GmbH, Landsberger Strasse 65, 82110 Germering

erklären in alleiniger Verantwortung, dass die Produkte „Cub Audio Test“ und seine Zubehörteile, mit der Artikelnummer 100360-CUB allen anwendbaren Grundlegenden Anforderungen der Richtlinien 2011/65/EU (RoHS), 2014/35/EU (Niederspannung), sowie der Richtlinie 2014/30/EU (EMV) entsprechen.

Alle angewandten harmonisierten und nationalen Normen und andere normative Dokumente sind in der Technischen Dokumentation (STED) aufgeführt.

Die Konformität wurde durch ein Konformitätsbewertungsverfahren gemäß den vorab genannten Richtlinien nachgewiesen. Diese Konformitätserklärung ist gültig ab Unterzeichnung.

ENGLISH

We

declare under our sole responsibility that the devices „Cub Audio Test“, including all its accessories, with article number 100360-CUB comply with all applicable Essential Requirements of Directives 2011/65/EU (RoHS), 2014/35/EU (Low Voltage) and 2014/30/EU (EMC).

All applied harmonised standards, national standards or other normative documents are listed in the “Technical Documentation” (STED).

The conformity was proven by conformity assessment procedure according to the directives mentioned above. This Declaration of Conformity is valid starting date of signature.

Germering, den 07.01.2020

Dr.-Ing. J. Oswald
Geschäftsführer
Managing Director
