# Supplementary material for: Rapid hearing threshold assessment with modified auditory brainstem response protocols in dogs
Source: Front Vet Sci. 2024 Mar 6;11:1358410. doi: 10.3389/fvets.2024.1358410 (PMC10951061; doi:10.3389/fvets.2024.1358410)
Supplement: Supplementary file 3 [file Presentation_1.pdf]

Supplementary Files

Supplementary file S1 Product information sheet (data sheet 1)
Supplementary file S2 Conformity declaration (data sheet 2)
